# Centromere-Specific Single-Copy Sequences of *Secale* Species

**DOI:** 10.3390/plants11162117

**Published:** 2022-08-15

**Authors:** Zijin Pan, Jie Luo, Zongxiang Tang, Shulan Fu

**Affiliations:** 1 College of Agronomy, Sichuan Agricultural University, Wenjiang, Chengdu 611130, China; 2 Provincial Key Laboratory for Plant Genetics and Breeding, Sichuan Agricultural University, Wenjiang, Chengdu 611130, China

**Keywords:** rye, centromere, single-copy sequence, 1BL.1RS translocation

## Abstract

Single-copy FISH analysis is a useful tool to physically locate a given sequence on chromosome. Centromeric single-copy sequences can be used to locate the position of centromere and disclose the subtle differences among different centromeres. Nine centromeric single-copy sequences 1R1, 3R1, 4R1, 4R2, 5R1, 5R2, 6R2, 6R3, and 7R1 were cloned from Kustro (*Secale cereale* L.). FISH analysis using these sequences as probes indicated that the signals of 1R1, 3R1, 4R1, 4R2, 5R1, 5R2, 6R1, 6R2, and 7R1 were located in the centromeric regions of rye 1R, 3R, 4R, 4R, 5R, 5R, 6R, 6R, and 7R chromosomes, respectively. In addition, for each of the centromeric single-copy sequences, high sequence similarity was observed among different *Secale* species. Combined with rye genomic sequence, single-copy FISH analysis indicated that the 1BL.1RS translocations in wheat cultivar CN17 and wheat line 20T363-4 contained the centromeric segment of 1R chromosome from 349,498,361 to 349,501,266 bp, and the 1BL.1RS translocations in the other two wheat cultivars did not contain this segment. The nine sequences are useful in determining the centromere location on rye chromosomes, and they have the potential to disclose the accurate structural differences of centromeres among the wheat-rye centric fusion translocation chromosomes; therefore, more centromeric single-copy sequences are needed.

## 1. Introduction

Cytogenetic methods, such as genomic in situ hybridization (GISH) and fluorescence in situ hybridization (FISH) have been widely used to study the genome structure and evolution of plant [1]. Among these modern cytogenetic approaches, the FISH analysis using single-copy DNA sequences as probes is useful in studying the chromosome rearrangement and evolution [2]. Single-copy genes were used as FISH probes to identify cucumber chromosomes and to study the homeologous relationship between *Cucumis anguria* and cucumber [3]. Moreover, this method was used to investigate the collinearity, chromosomal rearrangement, and homeologous relationship within the Triticeae [4,5,6,7,8,9,10]. These published reports provide comparative FISH maps of these plants and disclose several new chromosomal rearrangements of wheat [3,4,7,8]; however, the focus is on the chromosomal arms. In fact, this method can also be used to analyze the structure of centromere. Centromere is an important genetic locus on chromosome, which maintains the faithful transmission and the separation of a replicated chromosome to daughter cells. Although a functional centromere is determined by epigenetic mechanisms, centromere-specific DNA sequences are still the hallmark of centromeres. Tandem repeats and retrotransposons are the main components in centromeres. Few centromeric repeats are species-specific [11,12,13,14], and some exist in multiple species [15]. These repetitive DNA sequences were often used to investigate the centromere structure, especially that of whole-arm Robertsonian translocations. For example, centromeric repetitive sequences, such as CRW and pAWRC.1 of wheat and rye were often used to analyze the structure of the centromeres of wheat-rye Robertsonian translocations [16,17,18]. Rye is one of the most important alien sources of elite genes in wheat breeding [19]. To study the centromeric structure of rye chromosomes is beneficial to create wheat-rye translocation chromosomes. However, it is difficult to study the centromeric structure of rye chromosomes using repetitive DNA sequences due to their distribution characters and complexity. Possibly, to explore some centromere-specific single-copy sequences can help in resolving this problem since the physical sites of single-copy sequences on chromosomes are unique, and this can help in determining the positions of a given chromosomal segment accurately. In this study, some rye chromosome-specific centromere single-copy sequences were discovered.

## 2. Results

### 2.1. Cloning of Single-Copy Sequences

Nine sequences were amplified from the rye Kustro using the nine primer pairs (Table 1). Furthermore, primer pairs Primer-1R1 and Primer-3R1 amplified their target sequences from *Secale sylvestre* and *Secale strictum*, and Primer-4R1 and Primer-7R1 amplified their target sequences from *Secale strictum* (Table 2). A total of 15 single-copy sequences were cloned and sequenced (Table 2). The 15 sequences can be found in NCBI GenBank with the accession number (Table 2). These sequences had over 99% similarity to their corresponding sequences of rye Weining (Table 2). The sequences amplified by a primer pair from different *Secale* species were also highly similar (Figures S1–S4). Although these sequences were cloned from a few *Secale* species, they reflected their conservation.

### 2.2. Location of Single-Copy Sequences on Rye Chromosomes

**Figure 1:**
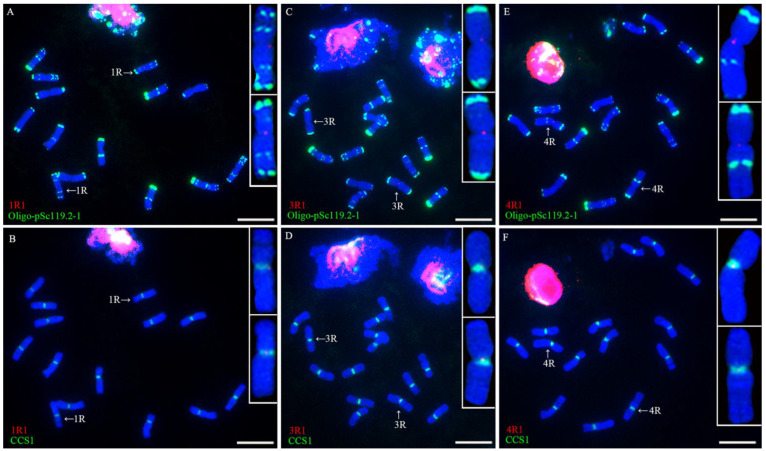
FISH analysis of root-tip metaphase chromosomes of rye Kustro using 1R1 (red), 3R1 (red), 4R1 (red), Oligo-pSc119.2-1 (green), and CCS1 (green) as probes. (**A**,**B**) A cell hybridized with 1R1, Oligo-pSc119.2-1, and CCS1. (**C**,**D**) A cell hybridized with 3R1, Oligo-pSc119.2-1, and CCS1. (**E**,**F**) A cell hybridized with 4R1, Oligo-pSc119.2-1, and CCS1. Scale bar: 10 μm.

**Figure 2:**
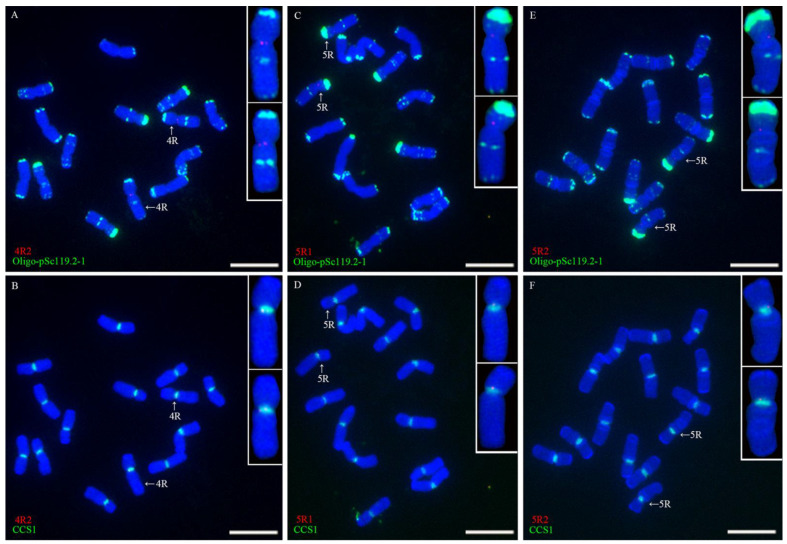
FISH analysis of root-tip metaphase chromosomes of rye Kustro using 4R2 (red), 5R1 (red), 5R2 (red), Oligo-pSc119.2-1 (green), and CCS1 (green) as probes. (**A**,**B**) A cell hybridized with 4R2, Oligo-pSc119.2-1, and CCS1. (**C**,**D**) A cell hybridized with 5R1, Oligo-pSc119.2-1, and CCS1. (**E**,**F**) A cell hybridized with 5R2, Oligo-pSc119.2-1, and CCS1. Scale bar: 10 μm.

**Figure 3:**
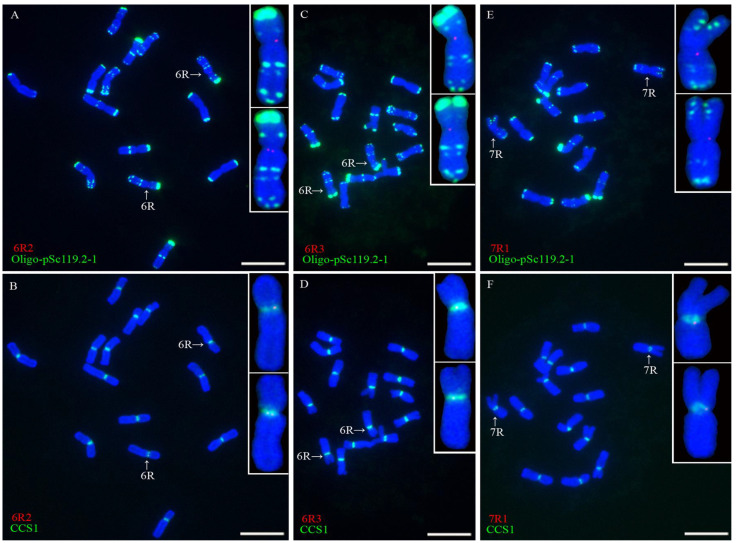
FISH analysis of root-tip metaphase chromosomes of rye Kustro using 6R2 (red), 6R3 (red), 7R1 (red), Oligo-pSc119.2-1 (green), and CCS1 (green) as probes. (**A**,**B**) A cell hybridized with 6R2, Oligo-pSc119.2-1, and CCS1. (**C**,**D**) A cell hybridized with 6R3, Oligo-pSc119.2-1, and CCS1. (**E**,**F**) A cell hybridized with 7R1, Oligo-pSc119.2-1, and CCS1. Scale bar: 10 μm.

The nine single-copy sequences cloned from rye Kustro were used as probes for the single-copy FISH analysis of the root-tip metaphase chromosomes of rye Kustro. The FISH analysis indicated that all the signals of the nine single-copy sequences appeared in the centromeric regions of their corresponding rye chromosomes. Each rye chromosome can be identified by the signal pattern of probe Oligo-pSc119.2-1. The signals of 1R1 only appeared in the centromeric region of 1R chromosome (Figure 1A,B), only the centromeric region of 3R chromosome contained the signal of 3R1 (Figure 1C,D), the signals of 4R1 and 4R2 only occurred in the centromeric region of 4R chromosome (Figure 1E,F and Figure 2A,B), 5R1 and 5R2 only produced signals in the centromeric region of 5R chromosome (Figure 2C–F), 6R1 and 6R2 produced signals in the centromeric region of 6R chromosome (Figure 3A–D), and the signal of 7R1 was only observed in the centromeric region of 7R chromosome (Figure 3E,F). In addition, the FISH signals of all the single-copy sequences were colocalized with CCS1 (Figure 1B,D,F, Figure 2B,D,F and Figure 3B,D,F). According to the dot FISH signals and the signal positions of these probes, it can be determined that these sequences are single-copy and centromeric, and they are chromosome-specific.

**Figure 4:**
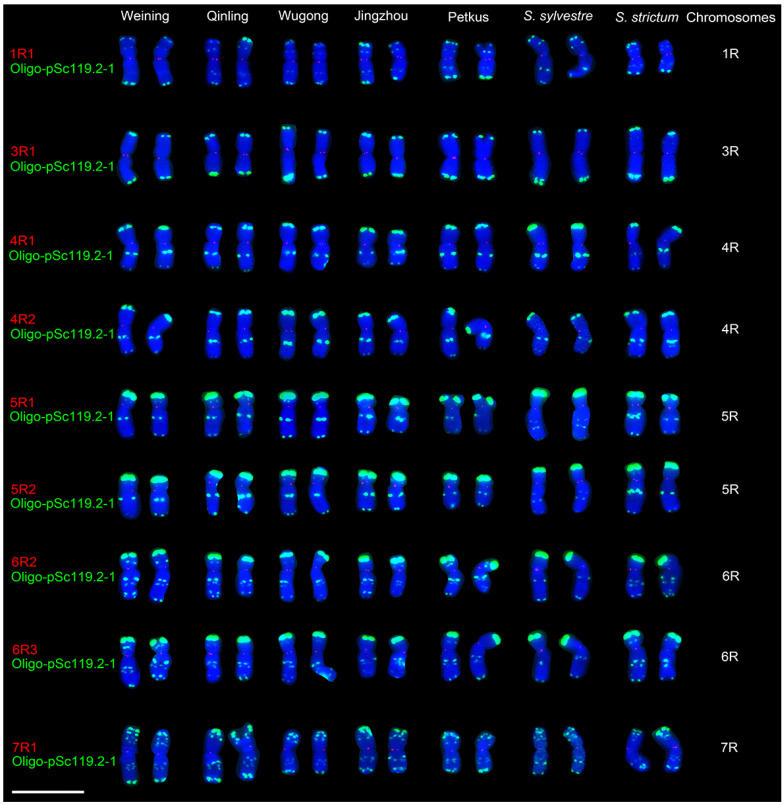
Cut-and-paste chromosomes of seven *Secale* species with the FISH signals of the nine single-copy sequences. Each rye chromosome can be identified by the signals of Oligo-pSc119.2-1 (green) and the signals of the nine single-copy sequences (red) are located in the centromeric regions. Scale bar: 30 μm.

In addition, the nine single-copy probes were used to analyze the root-tip metaphase chromosomes of the other five cultivated rye (Weining, Qinling, Wugong, Jingzhou, and Petkus) and two wild rye (*Secale sylvestre* and *Secale strictum*). The individual rye chromosome was judged by the signal pattern of the probe Oligo-pSc119.2-1. It can be noted that all the FISH signals of the nine probes were only located in the centromeric regions of their corresponding chromosomes (Figure 4). For instance, the signals of the probes 1R1 can only be observed in the centromeric regions of 1R chromosomes, and the other probes displayed similar results (Figure 4). These results indicated that these single-copy sequences were centromeric.

### 2.3. Different Centromeric Structure of Wheat-Rye 1BL.1RS Translocations

**Figure 5:**
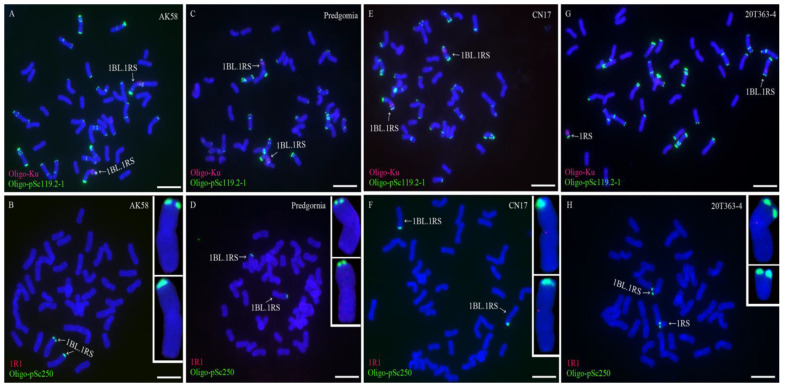
ND-FISH and single-copy FISH analyses of the root-tip metaphase chromosomes of AK58, Predgornia, CN17, and 20T363-4. (**A**,**C**,**E**,**G**) ND-FISH analysis using Oligo-Ku (red), Oligo-pSc119.2-1 (green) as probes. (**B**,**D**,**F**,**H**) The signal patterns of probe 1R1 (red) and oligo probe Oligo-pSc250 (green) in the four wheat-rye 1BL.1RS translocations. Scale bar: 10 μm.

The single-copy probe 1R1 was used to study the centromeric structure of wheat-rye 1BL.1RS translocation chromosomes in three wheat cultivars AK58, Predgornia, and CN17, and a wheat line 20T363-4. ND-FISH analysis using oligo probes Oligo-Ku and Oligo-pSc119.2-1 indicated that AK58, Predgornia, and CN17 contained a pair of wheat-rye 1BL.1RS translocations (Figure 5A,C,E). Line 20T363-4 contained a wheat-rye 1BL.1RS translocation chromosome and a 1RS arm (Figure 5G). Single-copy FISH analysis showed that no signals of probe 1R1 were observed on all the chromosomes (including the 1BL.1RS translocations) in AK58 and Predgornia (Figure 5B,D). The signals of this probe could only be observed in the centromeric region of 1BL.1RS chromosomes in CN17 and 20T363-4 (Figure 5F,H). Moreover, the 1RS arm in line 20T363-4 did not contain the signal of 1R1 (Figure 5H). The signals of probe 1R1 disclosed the different centromeric structures among different wheat-rye 1BL.1RS translocation chromosomes.

## 3. Discussion

Single-copy FISH analysis is a useful tool to physically locate a given sequence on chromosome [4,5,6,7,8,9,10]. Although functional centromere is determined by an epigenetical mechanism, centromeric repetitive DNA sequences are an important localizing marker of centromeres on chromosomes. However, it is difficult to determine the actual physical location of centromeres on chromosomes only by repetitive sequences since the sequence assembly in the centromere region is poor. In this study, combined with the assembled rye genomic sequences, single-copy FISH analysis was used to locate some single-copy sequences in centromeric regions of rye chromosomes. The results in this study are helpful in determining the centromere location on rye chromosomes. For example, the centromeric regions of 4R, 5R, and 6R chromosomes might be the segments from 409,233,133 to 414,936,603 bp, 237,421,693 to 245,445,320 bp, and 309,045,272 to 322,671,571 bp, respectively. 

Rapid evolution of centromeric repetitive sequences resulted in the centromere paradox [20]. In most of eukaryotic species, the same chromosome complement contains high similarity of centromere repetitive DNA sequences [21]. The divergence of centromeric satellites among the three subgenomes of common wheat (*Triticum aestivum* L.) was observed [22]. These results indicated that repetitive DNA sequences in centromeres are not chromosome-specific and are highly divergent. In potato, the centromeres of some chromosomes are composed by single-copy sequence, but their conservation was not reported [23]. In addition, these single-copy sequences in centromere are not chromosome-specific [23]. In this study, it was proved that the centromeres of rye chromosomes contain single-copy sequences. These single-copy sequences are chromosome-specific, and the centromere of each rye chromosome has its own conserved specific DNA sequence. Although the function of these single-copy sequences is unclear, they can be used to study the structure of centromere, especially the structure of the centromere in the wheat-rye centromeric fusion translocation chromosomes, such as wheat-rye 1BL.1RS translocation.

Wheat-rye 1BL.1RS translocation was formed by the centric fusion of the short arm of rye chromosome 1R and the long arm of wheat 1B chromosome. The centromere of 1BL.1RS translocation is a good model for studying the structure and function of centromere due to its hybrid character. The structure and function of the centromere of wheat-rye 1BL.1RS translocation have already been studied [16,17,18,24,25]. Immuno-FISH analysis indicated that the rye-derived centromere part is active in 1BL.1RS translocations [18], and this is different from the report that both the wheat- and rye-derived centromere parts displayed activity [17]. One of the reasons that caused the difference might be the different structure of centromere in 1BL.1RS translocations [18]. FISH analysis using centromere-specific repetitive DNA sequence indicated that wheat-rye translocations derived from centric breakage-fusion always contained hybrid centromeres [16,17]. It has already been reported that centric misdivision-fusion could result in centromeres with different structures [16,24,25]. In this study, the presence or absence of the FISH signal of single-copy sequences reflected the different centromeric structure in different 1BL.1RS translocations, and these different centromeres should come from different centric breakage-fusion events. In addition, at least it can be known that the 1BL.1RS translocations in CN17 and 20T363-4 contained the centromeric segment from 349,498,361 to 349,501,266 bp, and the 1BL.1RS translocations in the other two wheat cultivars did not contain this segment. Therefore, single-copy FISH has the potential to disclose the accurate structural differences among the centromeres of 1BL.1RS translocations.

## 4. Materials and Methods

### 4.1. Materials

Two wild rye *Secale sylvestre* Host and *Secale strictum* (C. Presl) C. Presl (PI 531829), and six cultivated rye (*Secale cereale* L.) including Kustro (PI 392065), Weining, Qinling, Wugong, Jingzhou, and Petkus (PI 330965), and three wheat-rye 1BL.1RS translocation cultivars AiKang 58 (AK58), Predgornia, and Chuannong 17 (CN17), and a 1BL.1RS line 20T363-4 were used in this study. PI 531829, PI 392065, and PI 330965 were kindly provided by the American Germplasm Resources Information Network (GRIN). The AK58 and Predgornia were kindly provided by Crop Research Institute, Sichuan Academy of Agricultural Sciences, China. The other materials were from our laboratory. The 1BL.1RS translocation cultivars Predgornia, CN17, and AK58 have been reported [26,27,28]. The 1BL.1RS translocation line 20T363-4 was identified from the progeny of common wheat (*Triticum aestivum* L.) Mianyang11 × rye (*Secale cereale* L.) Kustro [29].

### 4.2. Selection of Single-Copy Sequence

The cereal centromeric sequence CCS1 [30] and rye centromeric repeat pAWRC.1 [12] were aligned with the genomic sequence of Weining rye [31] to determine the approximate regions of centromeres. Then, single-copy sequences were selected from the genomic sequences in these regions on 1R to 7R chromosomes, and the process was carried out according to the method described by Zou et al. [32] with some modifications. Briefly, the candidate single-copy sequences of about 2000–3500 bp were selected every 5 Mb in the predicted centromere regions. The selected sequences were confirmed to be single-copy using BLAST tool in the B2DSC web server (http://mcgb.uestc.edu.cn/b2dsc, accessed on 6 May 2021) [33].

### 4.3. PCR Amplification and Sequence Cloning

Nine primer pairs were designed according to the candidate single-copy sequences from the rye Weining using Primer 3 software (version 4.0) (Table 1). Some progenies from common wheat × rye Kustro were obtained in our previous studies, and rye Kustro was used to clone the nine single-copy sequences, which is beneficial to the follow-up research. In addition, to test the conservation of the centromeric single-copy sequences, primer pairs Primer-1R1, Primer-3R1, Primer-4R1, and Primer-7R1 were randomly selected for cloning their corresponding sequences from wild species *Secale sylvestre* or *Secale strictum*. The PCR reaction mixture (50 μL total volume) contained 200 ng of template DNA, 3 μL of each primer with concentration of 10 μmol/L, and 42 μL 1.1 × T3 Super PCR Mix (Tsingke, Chengdu, China). PCR amplification was performed in a Coyote Bio PCR System (Coyote Bioscience, USA) using the following program: Pre-denaturation at 98 °C for 2 min, followed by 35 cycles of denaturation at 98 °C for 10 s, annealing at 55 to 60 °C depending on each primer for 10 s, extension at 72 °C for 1 min, and final extension at 72 °C for 2 min. The amplified products were electrophoresed in a 1.5% agarose gel in 1 × TAE buffer. The target sequences were cloned into TSINGKE pClone007 vector (Tsingke, Chengdu, China) and sequenced by the Tsingke Biotechnology Co., Ltd. (Chengdu, China). The cloned sequences were deposited in the GenBank Database. The similarity of these sequences were analyzed using DNAMAN (version 4.0, Lynnon Corp., Quebec, QC, Canada).

### 4.4. Single-Copy FISH Analysis

Single-copy FISH analysis was used to locate the single-copy sequences on rye chromosomes. The preparation of root-tip metaphase chromosomes was carried out according to the method described by Han et al. [34]. The procedures of single-copy FISH were performed as described by Zou et al. [32], with some modifications. Briefly, each of the single-copy probe was mixed with the oligonucleotide (oligo) probe Oligo-pSc119.2-1 [35] and (or) the centromeric repeated sequence CCS1 [30]. Slides were hybridized overnight at 55 °C and washed in 2 × SSC buffer for a few seconds at 55 °C first, then washed in ddH20 quickly at room temperature.

### 4.5. Non-Denaturing FISH (ND-FISH)

ND-FISH experiments were performed as described by Fu et al. [36]. The oligo probes used in this study were Oligo-pSc119.2-1 [35], Oligo-pSc250 [36], and Oligo-Ku [Xiao et al. 2017]. Oligo-pSc119.2-1 can be used to identify individual rye and wheat chromosomes [36]. Oligo-Ku and Oligo-pSc250 were rye-specific and these probes combined with Oligo-pSc119.2-1 can be used to identify wheat-rye 1BL.1RS translocation chromosomes [36,37].

## 5. Conclusions

In this study, nine centromere-specific single-copy sequences of rye were found. These sequences are useful in determining the centromere location on rye chromosomes. In addition, they have the potential to disclose the accurate structural differences of centromeres among the wheat-rye centric fusion translocation chromosomes; therefore, more centromeric single-copy sequences are needed.

## Figures and Tables

**Table 1:** Primer pairs for cloning the centromeric single-copy sequences.

Name of Primer	Sequence of Primer (5′-3′)	Target Region of Amplification (bp) *
Primer-1R1	F: GGGTGATTGGCATATCTCGT R: GCAGCCACAGGTATCTCGTT	1R: 349,498,361 to 349,501,266
Primer-3R1	F: ACATTGGGTCTCTGCGTCAG R: AGAACATCTCAATTCGTGGTGC	3R: 517,650,456 to 517,652,830
Primer-4R1	F: CAGACAGGCGAGCAGGATAG R: CTCTTCCGCAACAGCTACCT	4R: 409,233,133 to 409,235,918
Primer-4R2	F: GCTCGCTCTCTTACCCTTCC R: TGTTCTCACAGGCTTTTTCTCA	4R: 414,933,891 to 414,936,603
Primer-5R1	F: CTAGGCAGCTGGGTAATGCG R: CTCCTCTCCCCTAACCCTCC	5R: 237,421,693 to 237,423,703
Primer-5R2	F: CGCGATCCCCATCTCTGTTT R: GTCGCCTCACCTTACGCTTT	5R: 245,442,755 to 245,445,320
Primer-6R2	F: TGCCCAGACCAGCTAGACTA R: TACAACAGCAACCCGAGCAA	6R: 309,045,272 to 309,048,228
Primer-6R3	F: GTCTTGCAAATTGGTTCATGAGA R: CACTACAAGATACCAACTCCA	6R: 322,669,622 to 322,671,571
Primer-7R1	F: AGTGAAGTTCCCGTTGGTCA R: TCCAGCTGTTGAACCATCCA	7R: 468,542,502 to 468,545,143

* The left number indicates the position of the first base of the forward primer in the corresponding chromosome of rye Weining, and the right number indicates the position of the last base of the reverse primer in the corresponding chromosome of rye Weining.

**Table 2:** The information of the cloned centromeric single-copy sequences.

Name of Single-Copy Sequences	Length of Sequence (bp)	Source of Sequence	Location on Chromosome	GenBank Accession Number	Similarity with Its Corresponding Sequence in Weining (%)
1R1	2906	Kustro	centromere of 1R	ON557265	100.00
SL-1R1	2900	*Secale sylvestre*		ON557267	99.21
SD-1R1	2914	*Secale strictum*		ON557268	99.86
3R1	2373	Kustro	centromere of 3R	ON557271	99.92
SL-3R1	2382	*Secale sylvestre*		ON557272	99.16
SD-3R1	2374	*Secale strictum*		ON557273	99.96
4R1	2787	Kustro	centromere of 4R	ON557274	99.86
SD-4R1	2786	*Secale strictum*		ON557275	99.93
4R2	2713	Kustro		ON557276	99.78
5R1	2011	Kustro	centromere of 5R	ON557277	99.65
5R2	2566	Kustro		ON557278	99.84
6R2	2957	Kustro	centromere of 6R	ON557279	99.80
6R3	1950	Kustro		ON557280	99.69
7R1	2642	Kustro	centromere of 7R	ON557281	99.85
SD-7R1	2642	*Secale strictum*		ON557282	99.89

## Data Availability

The materials used in this study are available on request from the corresponding author. The sequences of primers used in this study can be obtained from Table 1. The cloned sequences can be obtained from the GenBank Database.

## Supplementary Materials

The following supporting information can be downloaded at: https://www.mdpi.com/article/10.3390/plants11162117/s1. Figure S1: Multiple alignment of the sequences amplified by the primer pair Primer-1R1; “original-1R1” indicates the sequence of rye Weining used to design the Primer-1R1; “1R1”, “SL-1R1”, and “SD-1R1” indicate the sequences from rye Kustro, *S. sylvestre,* and *S. strictum*, respectively. Figure S2: Multiple alignment of the sequences amplified by the primer pair Primer-3R1; “original-3R1” indicates the sequence of rye Weining used to design the Primer-3R1; “3R1”, “SL-3R1”, and “SD-3R1” indicate the sequences from rye Kustro, *S. sylvestre,* and *S. strictum*, respectively. Figure S3: Multiple alignment of the sequences amplified by the primer pair Primer-4R1; “original-4R1” indicates the sequence of rye Weining used to design the Primer-4R1; “4R1” and “SD-4R1” indicate the sequences from rye Kustro and *S. strictum*, respectively. Figure S4: Multiple alignment of the sequences amplified by the primer pair Primer-7R1; “original-7R1” indicates the sequence of rye Weining used to design the Primer-7R1; “7R1” and “SD-7R1” indicate the sequences from rye Kustro and *S. strictum*, respectively.
