# Supplementary material for: Centromere-Specific Single-Copy Sequences of Secale Species
Source: Plants (Basel). 2022 Aug 15;11(16):2117. doi: 10.3390/plants11162117 (PMC9414614; doi:10.3390/plants11162117)
Supplement: Supplementary file 1 [file plants-11-02117-s001.zip › plants-1855698-supplymentary.pdf]

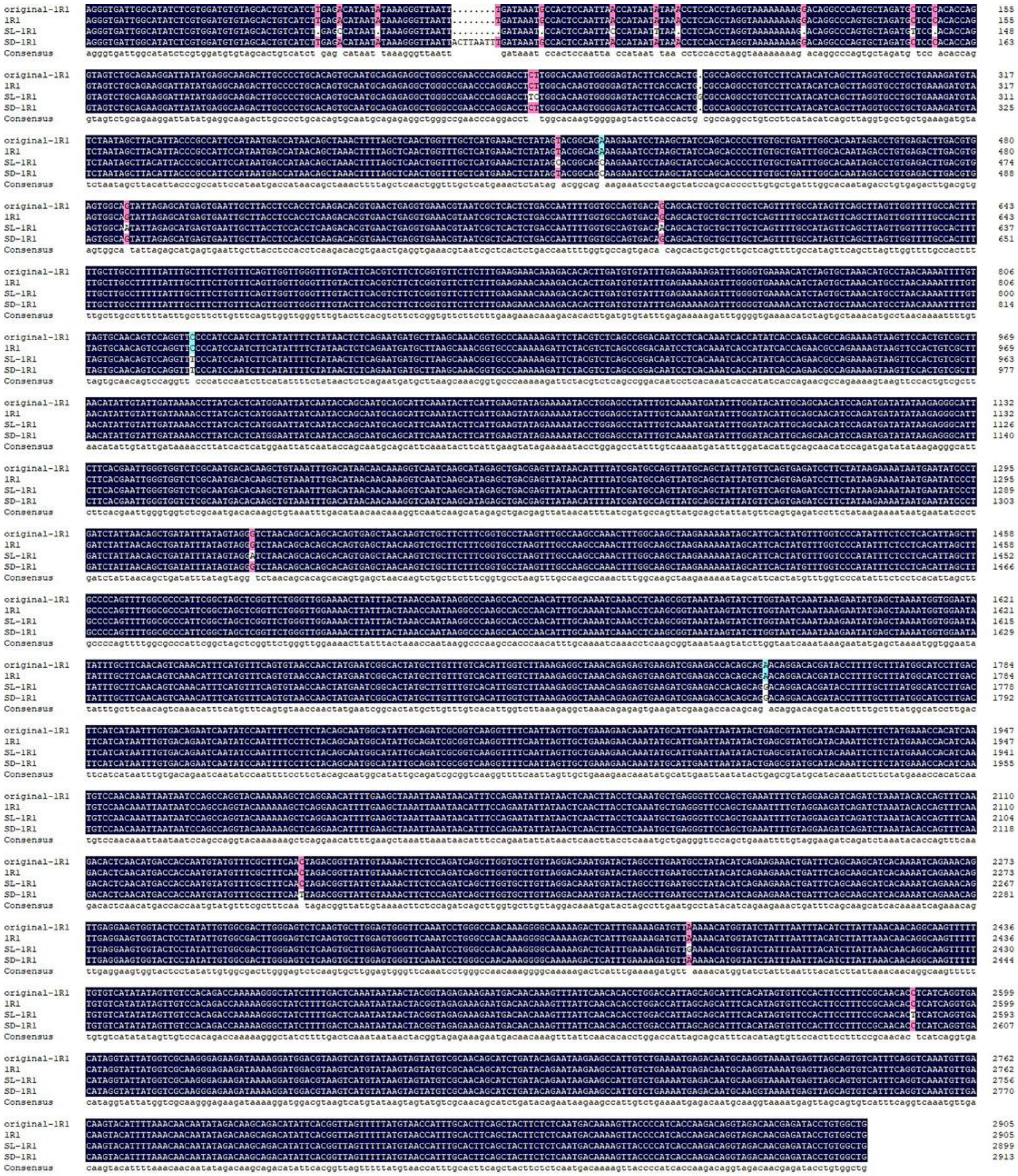

Figure S1. Multiple alignment of the sequences amplified by the primer pair Primer-1R1; “original-1R1” indicates the sequence of rye Weining used to design the Primer-1R1; “1R1”, “SL-1R1”, and “SD-1R1” indicate the sequences from rye Kustro, *S. sylvestre,* and *S. strictum*, respectively.

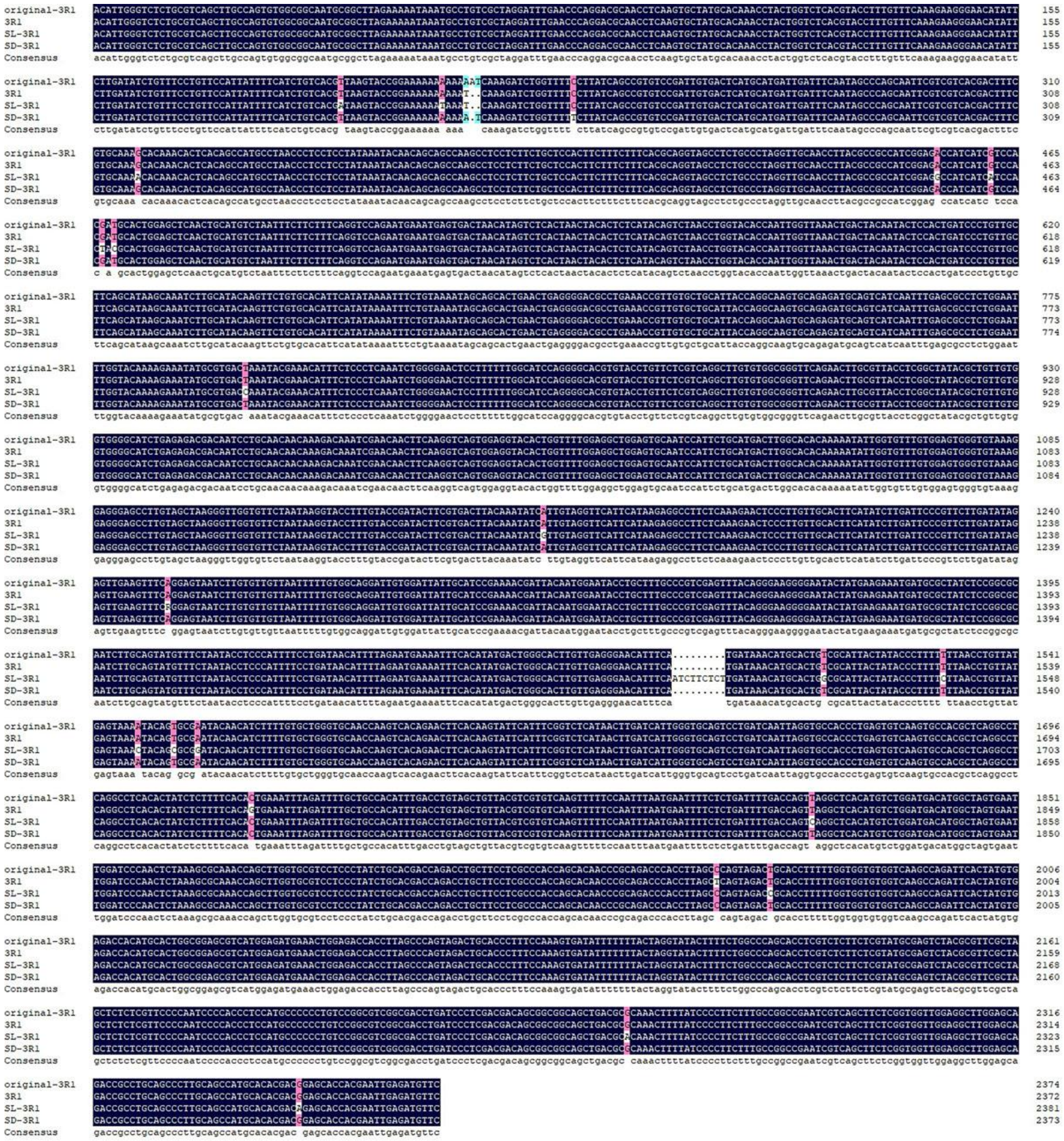

Figure S2. Multiple alignment of the sequences amplified by the primer pair Primer-3R1; "original-3R1" indicates the sequence of rye Weining used to design the Primer-3R1; "3R1", "SL-3R1", and "SD-3R1" indicate the sequences from rye Kustro, *S. sylvestre*, and *S. strictum*, respectively.

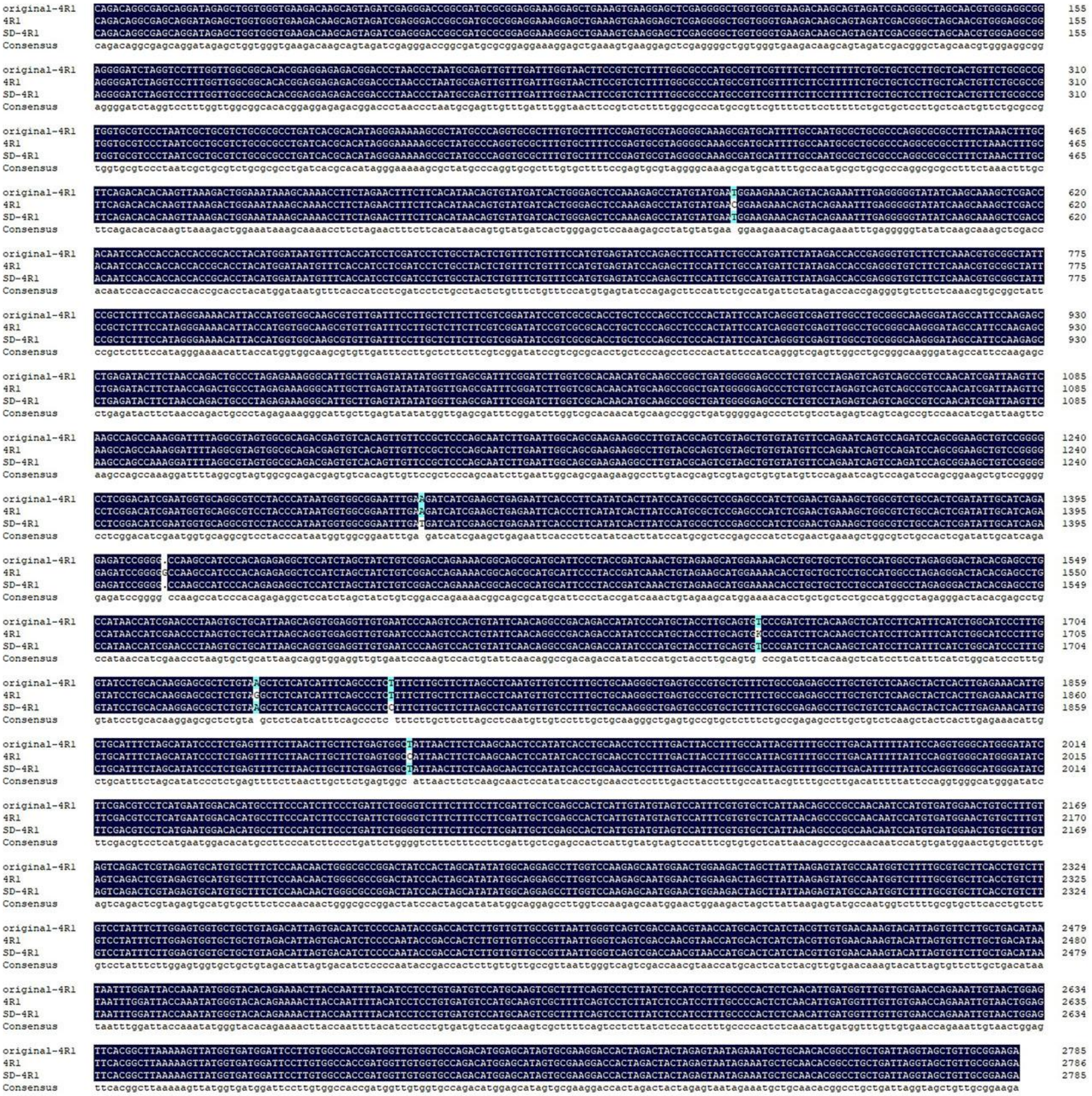

Figure S3. Multiple alignment of the sequences amplified by the primer pair Primer-4R1; "original-4R1" indicates the sequence of rye Weining used to design the Primer-4R1; "4R1" and "SD-4R1" indicate the sequences from rye Kustro and *S. strictum*, respectively.

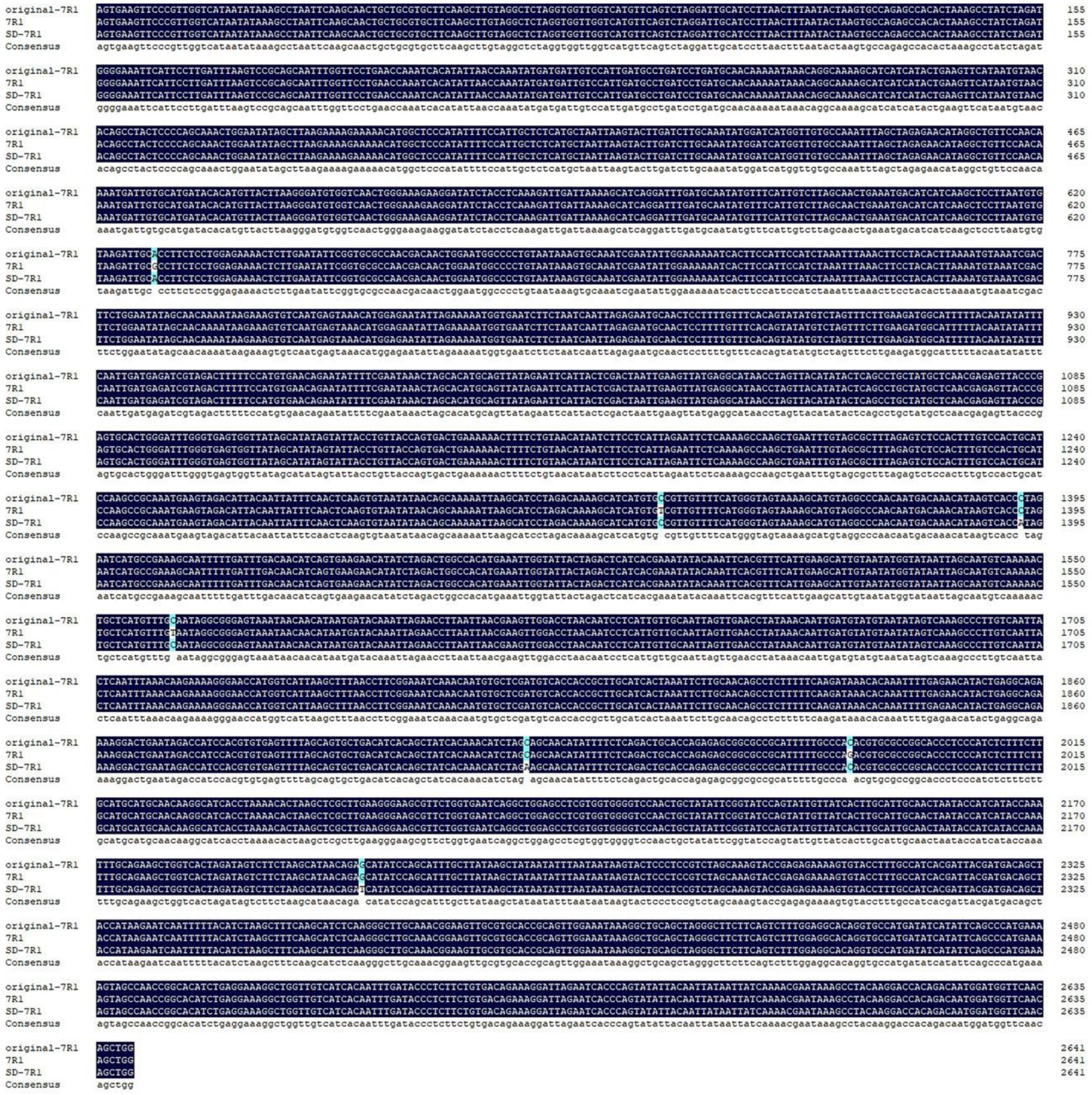

Figure S4. Multiple alignment of the sequences amplified by the primer pair Primer-7R1; “original-7R1” indicates the sequence of rye Weining used to design the Primer-7R1; “7R1” and “SD-7R1” indicate the sequences from rye Kustro and *S. strictum*, respectively.
